# The effects of small interfering RNA–targeting tissue factor on an in vitro model of neovascularization

**Published:** 2013-06-11

**Authors:** Wenyan Peng, Ying Yu, Tiejun Li, Yuanyuan Zhu, Hui Chen

**Affiliations:** 1 Eye Institute, Affiliated Hospital of Nantong University, Nantong, China; 2 Department of Life Science Center, Biomics Biotechnologies Co. Ltd, Nantong, China; 3 State Key Laboratory of Ophthalmology, Sun Yat-sen University, Zhongshan Ophthalmic Center, Guangzhou, China

## Abstract

**Purpose:**

Tissue factor (TF) plays an important role in neovascularization (NV). This study aimed to determine whether small interfering RNA–targeting TF (TF-siRNA) could knock down TF expression and inhibit cell proliferation, cell migration, and tube formation in an in vitro model of NV.

**Methods:**

Lipopolysaccharide (LPS) was used to stimulate human umbilical vein endothelial cell (HUVEC) lines to express TF and mimic certain phenotypes of NV in vitro. HUVECs were transfected with TF-siRNAs and control siRNAs using Lipofectamine^TM^ 2000. The inhibitory effect of the siRNAs on the expression of TF mRNA and protein was evaluated by quantitative reverse transcriptase polymerase chain reaction (RT-qPCR) and western blot analysis. The effects on the cell viability, migration, and tube formation of siRNA-treated cells were examined by MTT assay, wound-healing assay, and Matrigel-induced capillary tube formation.

**Results:**

Lipopolysaccharide treatment increased the expression of TF. TF**-**siRNAs effectively knocked down TF expression, with the most efficient TF-siRNA reducing 78.9% of TF expression. TF protein was also notably curtailed by TF-siRNA. The MTT and wound-healing assays showed that the TF-siRNA substantially inhibited the proliferation and migration of HUVECs. Tube formation was decreased by 47.4% and 59.4% in cells treated with the TF-siRNA and vascular endothelial growth factor–siRNA, respectively, compared with the blank control.

**Conclusions:**

TF-siRNA can knockdown TF expression and inhibit cell proliferation, migration, and tube formation in vitro. TF-siRNA may provide a novel therapeutic candidate for NV-related diseases.

## Introduction

Choroidal neovascularization (CNV) refers to the pathological growth of new blood vessels from the choroid [1]. It is a major feature in the advanced stage of wet age-related macular degeneration (AMD), pathological myopia, and many other ocular diseases. Exudation and hemorrhage are often associated with CNV; these can cause serious damage of the central visual acuity and lead to irreversible blindness. The mechanism of CNV is still far from being completely understood. In general, CNV can be induced through an imbalance between proangiogenic and antiangiogenic factors in local retina tissue; this is triggered by aging, oxidative stress, and the inflammatory response [2-4].

Vascular endothelial growth factor (VEGF) is one of the proangiogenic factors in the CNV microenvironment [5-7]. It can promote augmentation and migration of vascular endothelial cells, increase the permeability of vessels, and induce capillary lumen formation. High expression of VEGF has been found in the aqueous humor, vitreous, retinal pigment epithelium, and basement membrane of CNV patients [8-11], as well as in endothelial cells and macrophages in an animal model of CNV [12-15]. The neutralization of VEGF has become a mainstay of CNV treatment [16-19]. Beyond its potent angiogenic effect, VEGF also has neuroprotective properties [20,21]. Studies have demonstrated that VEGF promotes neurite regrowth of axotomized retinal ganglion cells [22,23]. Therefore, excessive inhibition of VEGF may present a risk for retinal ganglion cell damage, making it necessary to find a safe and effective approach to interfere with CNV other than the inhibition of VEGF.

Recent studies have suggested that tissue factor (TF) may play an important role in the formation of CNV [24,25]. High expression of TF was found in retinal cells and tissues of an AMD animal model with CNV, as well as in an in vitro retinal pigment epithelium model of inflammation [24]. In the human retina, a 32-fold increase of TF messenger RNA (mRNA) expression was detected in AMD macular lesions compared with the normal macula. TF protein expression was also enhanced in the human AMD macula [24]. Furthermore, inflammation-active human CNV showed much more intense TF reactivity than inflammation-inactive CNV [26].

TF is a member of the Type II cytokine receptor superfamily and the receptor of clotting factor VII (FVII)/active FVII (FVIIa) and functions in fibrin formation [26,27]. Normal retinal cells were low in TF expression [25]. The TF/FVIIa complex promotes inflammation and angiogenesis through a protease-activated receptor that regulates intracellular signal transduction. Meanwhile, inflammation may increase the expression of TF. Conversely, high expression of TF may increase the expression of cytokines such as interleukin (IL)-1β, IL-6, IL-8, and MIP-2a/CXCL2a, which are associated with inflammation [27,28]. The high expression of TF present in CNV suggests that inhibiting TF might prevent the formation of CNV, and could be an effective therapy for CNV-related diseases. Potential strategies for inhibiting TF include the deactivation of TF through neutralization or knockdown of its expression. Earlier studies have demonstrated that Icon, a synthetic molecule composed of FVII conjugated to the Fc domain of an antibody [29-31], binds to TF on pathologic blood vessels in the CNV [32,33], thereby activating the immune response mediated by natural killer cells and complement [34] and destroying the CNV.

Small interfering RNA (siRNA) has emerged as a potential therapeutic method for various diseases [35]. siRNA-targeting TF (TF-siRNA) was successfully used to silence the expression of TF in lung adenocarcinoma A549 cell lines and to reduce tumor growth and metastasis [36]. The present study aimed to test whether TF-siRNA could knock down TF gene expression and regulate cell proliferation, cell migration, and tube formation in human umbilical vein endothelial cell (HUVEC) lines under lipopolysaccharide (LPS) stimulation, which is widely used as an in vitro model of NV.

## Methods

### Preparation of small interfering RNA

Three pairs of TF-siRNA (TF-siRNA-1, TF-siRNA-2, and TF-siRNA-3), one pair of VEGF-siRNA, and one pair of nonspecific-siRNA (NS-siRNA) were designed and synthesized by Biomics Biotechnologies Co. Ltd., Nantong, China. All siRNAs were 21-nucleotide dsRNA oligos with a two-nucleotide (TT) overhang at the 3′ end. The siRNA sequences are listed in Table 1.

**Table 1:** The siRNAs used in the experiments.

Number	Name	Sequence (5′→3′)
TF-siRNA-1	F	GGCAGCAUAUAAUUUAACUdTdT
	R	AGUUAAAUUAUAUGCUGCCdTdT
TF-siRNA-2	F	GAGCCUCUGUAUGAGAACUdTdT
	R	AGUUCUCAUACAGAGGCUCdTdT
TF-siRNA-3	F	GGCAAGGACUUAAUUUAUAdTdT
	R	UAUAAAUUAAGUCCUUGCCdTdT
VEGF-siRNA	F	CUGAGUUUAAAAGGCACCCdTdT
	R	GGGTGCCUUUUAAACUCAGdTdT
non-specific-siRNA	F	UUCUCCGAACGUGUCACGUdTdT
(NS-siRNA)	R	ACGUGACACGUUCGGAGAAdTdT

### Cell culture

The HUVECs were purchased from American Type Culture Collection (ATCC, Manassas, VA) and were maintained in Dulbecco’s Modified Eagle’s Medium (DMEM; Gibco BRL, Grand Island, NY) supplemented with 10% fetal bovine serum (FBS) and 100 U/ml penicillin-streptomycin mixture (Gibco BRL) at 37 °C and 5% CO_2_ in a humidified chamber.

### Transfection of small interfering RNA–targeting tissue factor

HUVECs cells (2×10^5^ cells/well) were plated in six-well plates and allowed to grow overnight. siRNA (100 pmol) and 5 μl Lipofectamine™ 2000 (Invitrogen, Carlsbad, CA) were diluted in DMEM to a total volume of 250 μl. The diluted siRNA and Lipofectamine™ 2000 were mixed and incubated at room temperature for 20 min to generate the transfection mixture. The cells were washed with serum-free DMEM medium, and then the transfection mixture was added to the six-well plates and incubated for 5 h.

### Quantitative reverse transcriptase polymerase chain reaction

Total RNA was isolated from HUVECs using TRIzol (Invitrogen, Carlsbad, CA). Quantitative reverse transcriptase polymerase chain reaction (RT-qPCR) was performed for TF using the SYBR green method with an iQ^TM^5 Real-Time PCR Detection System (BIO-RAD, Hercules, CA). Cycling conditions for amplification were: 95 °C for 5 min, followed by 45 cycles at 95 °C for 30 s and 72 °C for 30 s. The Ct values were recorded and the specificity of amplification was evaluated by melting curve analysis. The RT-qPCR primers are listed in Table 2. The relative gene expression levels were calculated using the comparative Ct(ΔΔCt) method, where the relative expression was calculated as 2^-ΔΔCt, and Ct represents the threshold cycle [37], using *GAPDH* as the reference gene.

**Table 2:** The primers used in the experiments.

Number	Name	Sequence (5′→3′)
TF	F	TGCACCACCAACTGCTTAGC
	R	GGCATGGACTGTGGTCATGAG
GAPDH	F	CCGAACAGTTAACCGGAAGA
	R	TCAGTGGGGAGTTCTCCTTC

### Western blot analysis

Cells were grouped as in the RT-qPCR studies. The cells were lysed and proteins were separated using 10% sodium dodecyl sulfate–polyacrylamide gel electrophoresis. After electrophoresis, proteins were electrotransferred onto PVDF membranes (Millipore, Billerica, MA); after the transfer, they were blocked for 2 h in blocking buffer containing 5% nonfat dry milk. Membranes were incubated overnight at 4 °C with a TF primary antibody (sc-80952, Santa Cruz Biotechnology, Inc., Santa Cruz, CA), then washed and incubated with a horseradish peroxidase–conjugated secondary antibody (Santa Cruz Biotechnology Inc.) in Tris-Buffered Saline and Tween 20 (TBST) for 2 h at room temperature. Horseradish peroxidase–conjugated β-actin (sc-47778, Santa Cruz Biotechnology, Inc.) was used as internal control. Detection was performed using an ECL chemiluminescence kit (Pierce, Rockford, IL). The film was scanned using ImageQuant software (Molecular Dynamics, Sunnyvale, CA).

### Cell proliferation assay

Cell proliferation was measured using the MTT assay following the manufacturer’s instructions. Briefly, cells were seeded on a 96-well cell culture cluster (Corning Inc., Corning, NY) at a concentration of 10^4^cells/well in a volume of 100 µl, and grown overnight. Cell proliferation was measured every 24 h for 72 h. At the end of each time point, 20 μl of 5 mg/ml MTT (Sigma, St. Louis, MO) was added to each well. Four hours later, 150 μl of dimethylsulfoxide was added to the MTT-treated wells for 10–15 min and the absorption was determined at 570 nm using an automated plate reader. Each experimental condition was performed in triplicate.

### Wound-healing assay

HUVECs were transfected with siRNAs in a 24-well plate. After 48 h, the cells were grown to confluence and scratched with sterile 200 μl pipette tips. Plates were washed twice with PBS to remove detached cells and incubated in the complete growth medium without FBS. Cells migrated into the wounded area, and photographs were taken immediately (0 h) and at 24 h.

### Matrigel-induced capillary tube formation

This assay was performed by the method as previously described [38]. Matrigel^TM^ Basement Membrane Matrix (cat 356,234, BD Biosciences, San Jose, CA) was added to 24-well plates to a total volume of 200 μL in each well. Plates were stored at 37 °C for 30 min to form a gel layer. After gel formation, 500 μl HUVECs (10^5^ cells) in a medium containing 10% FBS was placed in each well. After incubation for 5 h or 12 h, the cells were observed with an inverted phase-contrast microscope (Olympus, Shinjuku, Tokyo, Japan) and photographed. The number of branching points was counted. Only points generating at least three tubules were counted.

### Statistical analysis

All data were shown as mean±standard deviation (SD). Statistical significance was determined by Student *t* test using the SPSS 17.0 software package. The level of statistical significance was set at p<0.05.

## Results

### Effect of lipopolysaccharide on cellular viability

**Figure 1:**
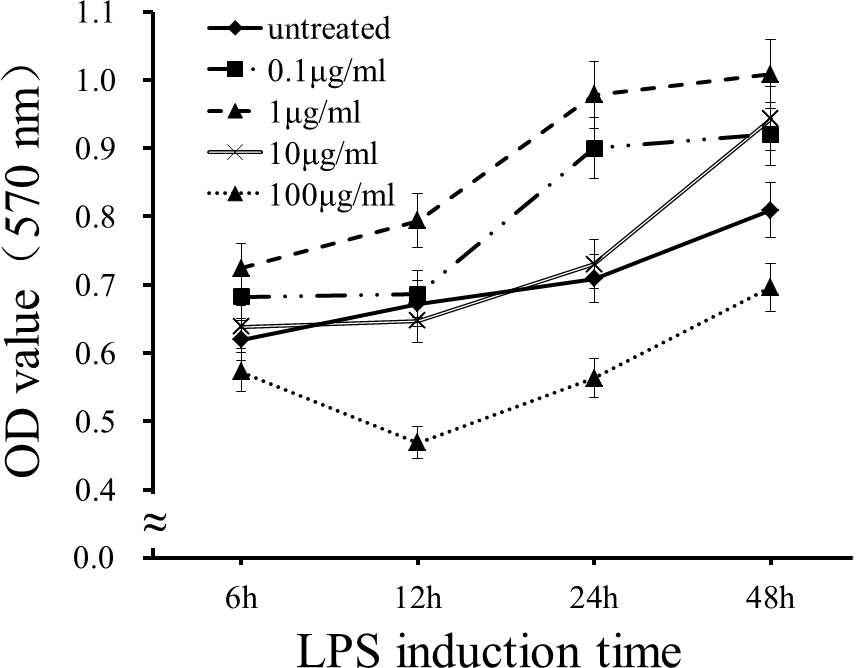
The effect of lipopolysaccharide on human umbilical vein endothelial cell viability by MTT assay. The cell viability from three independent experiments are expressed as mean ±standard deviation (SD; n=3).

To avoid the potential cytotoxic effect of LPS, the MTT test (described above) was performed to determine the safe concentration range of LPS (0 μg/ml, 0.1 μg/ml, 1 μg/ml, 10 μg/ml, 100 μg/ml) in HUVECs. The results showed that LPS at 10 μg/ml had no significant effect on HUVECs viability (p>0.05, Figure 1), and this dose was used for the following experiments.

### Downregulation of the tissue factor expression in lipopolysaccharide-induced human umbilical vein endothelial cells after small interfering RNA–targeting tissue factor transfection

A fivefold increase of TF mRNA expression was detected in HUVECs after 10 μg/ml LPS stimulation compared with untreated HUVECs. LPS treatment consistently increased TF transcript and protein expression (data not shown).

**Figure 2:**
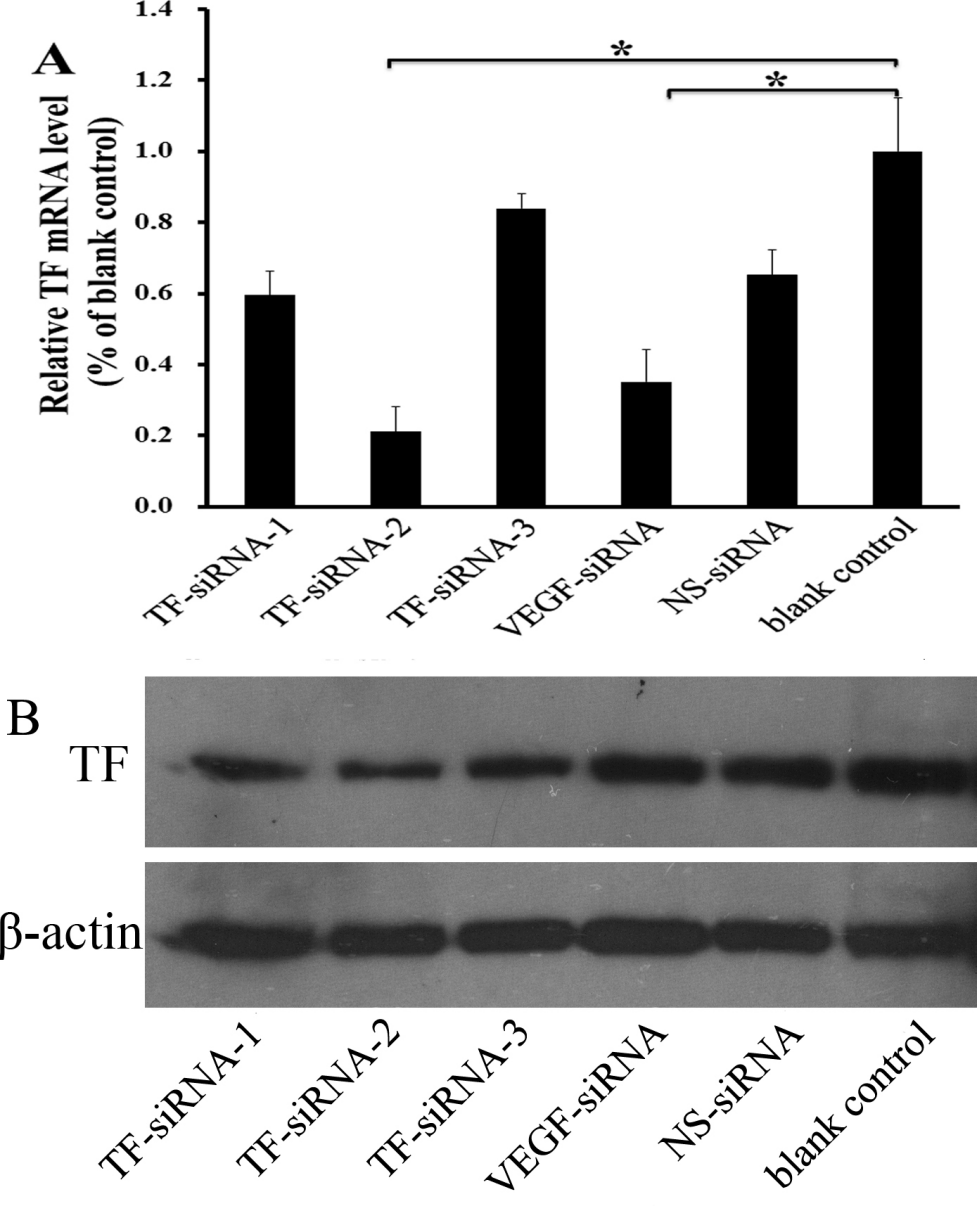
Effects of small interfering RNA–targeting tissue factor on tissue factor knockdown in the in vitro model of neovascularization. **A**: Tissue factor (TF) messenger RNA (mRNA) expression levels in human umbilical vein endothelial cells (HUVECs) after lipopolysaccharide (LPS) stimulation at 24 h, after 5 h of exposure to various small interfering RNAs (siRNAs). Relative quantification of TF mRNA expression is shown as mean±standard deviation (SD) of three independent experiments; *, p<0.01. **B**: Representative western blots show the TF protein expression levels at 24 h, after 5 h exposure to various siRNAs. β-actin was the internal loading control.

HUVECs were transiently transfected with the siRNAs after LPS stimulation. As shown in Figure 2A,B, TF-siRNA transfection significantly reduced the amount of TF mRNA after 24 h of transfection. The levels of TF mRNA in the TF-siRNA-1, TF-siRNA-2, and TF-siRNA-3 treated groups were inhibited by 40.5%, 78.9%, and 16.0%, respectively, compared with the control group, and by 64.8% in the VEGF-siRNA group. TF-siRNA-2 was most efficient in knocking down TF mRNA expression. The TF protein level was also lower in the TF-siRNA-2 treated group compared to both the blank control group and the NS-siRNA treated group.

### Inhibition of cell proliferation by small interfering RNA–targeting tissue factor–2

**Figure 3:**
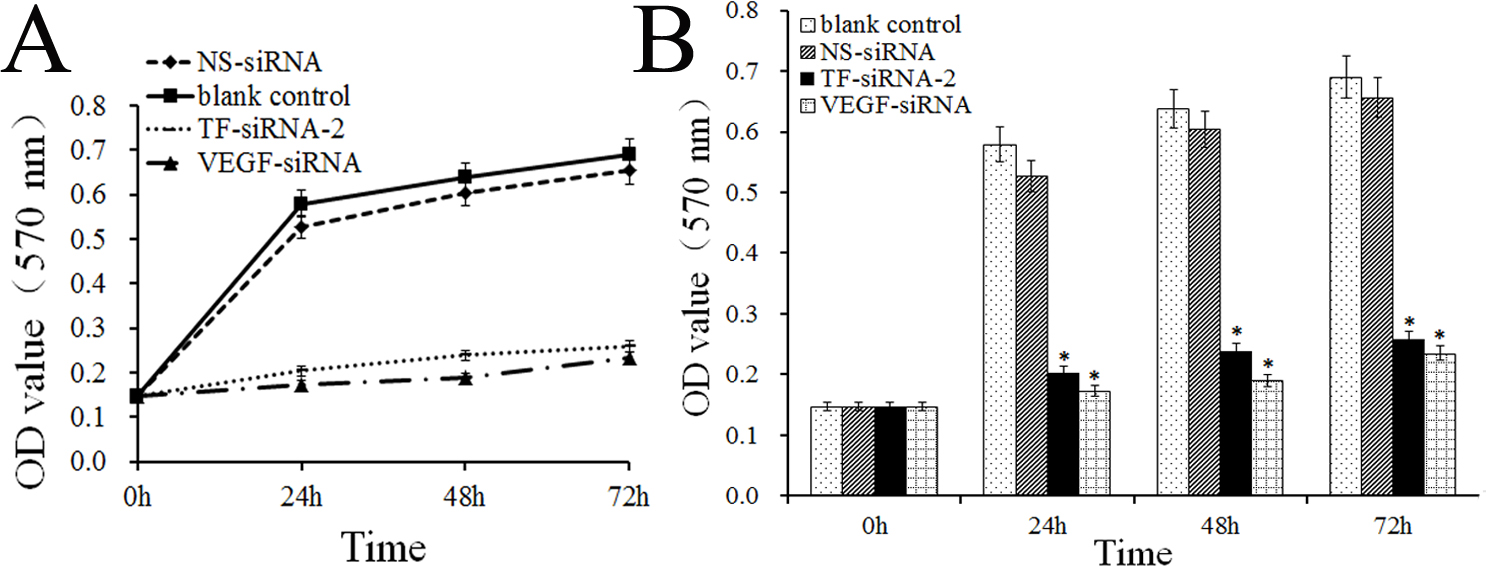
Inhibition of cell proliferation of human umbilical vein endothelial cells by small interfering RNA–targeting tissue factor–2. The cell growth of small interfering RNA–targeting tissue factor (TF-siRNA)-2 and vascular endothelial growth factor (VEGF)-siRNA–transfected human umbilical vein endothelial cells (HUVECs) was significantly attenuated in a time-dependent manner compared with the blank control group. *, p<0.01 versus blank control and nonspecific (NS)-siRNA.

As shown in Figure 3, after 24–72 h transfection of the TF-siRNA-2 into HUVECs, cell proliferation was remarkably inhibited in a time-dependent manner when compared with the blank control and the NS-siRNA treated group. Inhibition of cell proliferation began at 24 h post-transfection. In addition, the assay revealed identical effects to those of the VEGF-siRNA.

### Attenuation of the migration/invasion ability by small interfering RNA–targeting tissue factor–2

**Figure 4:**
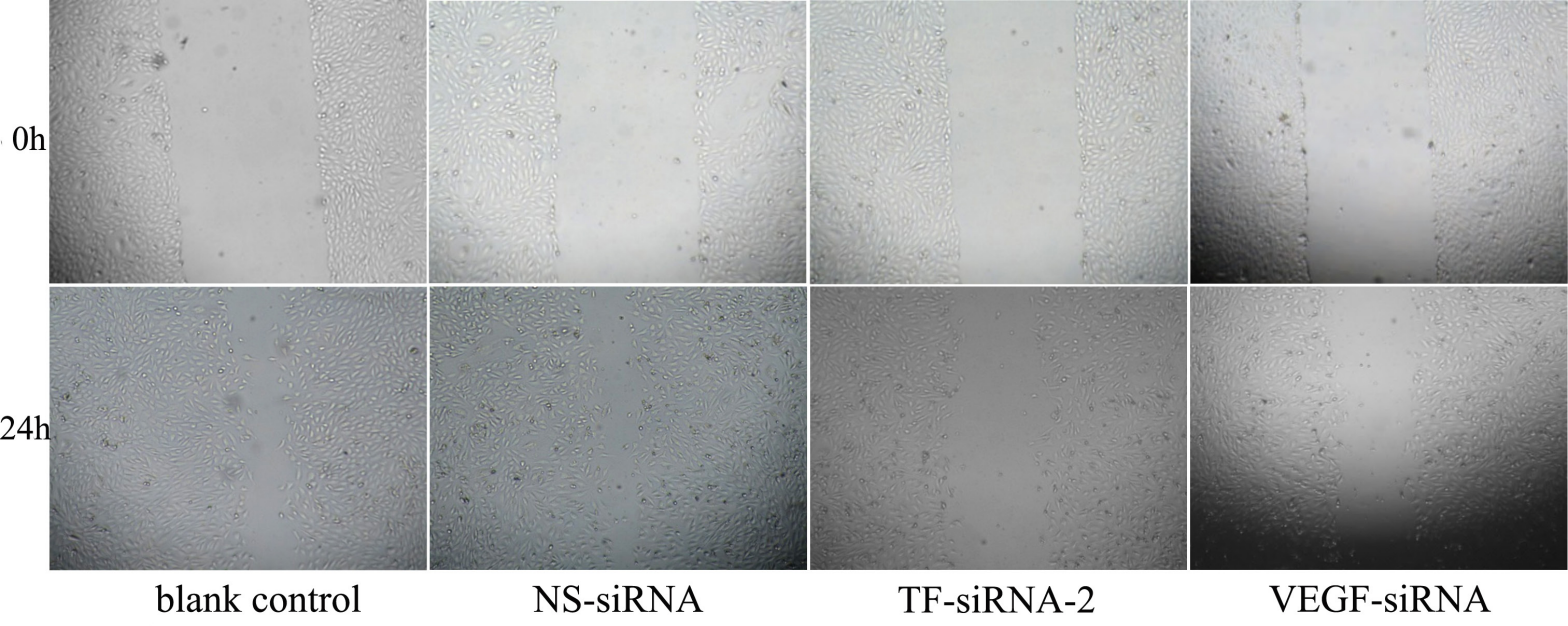
Attenuation of migration ability of human umbilical vein endothelial cells by small interfering RNA–targeting tissue factor–2. Representative images at 0 h and 24 h of the wound-healing assay are shown (×4).

Figure 4 shows that the cells in TF-siRNA-2 group demonstrate an attenuated capacity of impaired migration when compared to the blank control and the NS-siRNA–treated group.

### Effects of small interfering RNA–targeting tissue factor–2 on tube formation

**Figure 5:**
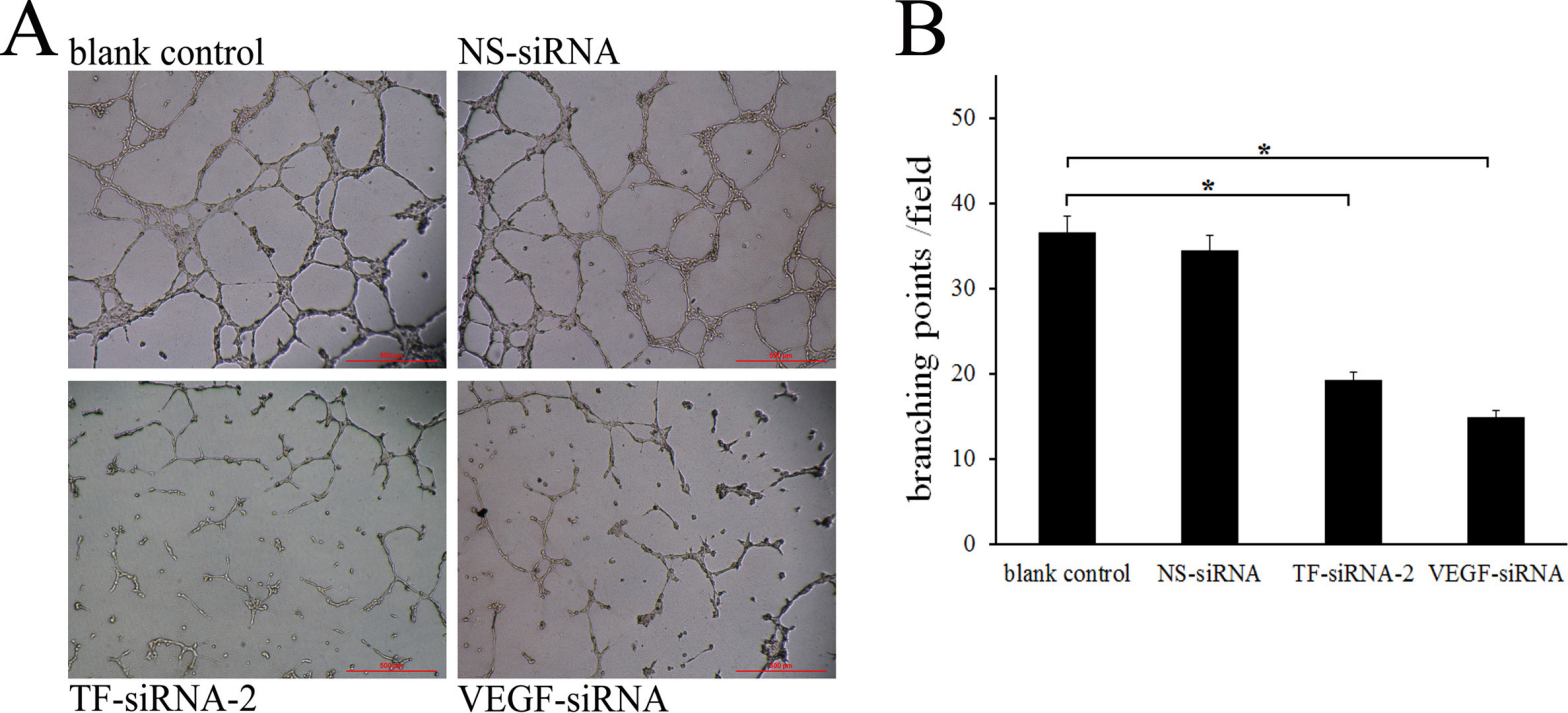
Effect of small interfering RNA–targeting tissue factor–2 on tube formation. Endothelial cell network formation was assessed using a Matrigel assay. **A**: Representative photographs of each group are shown (bar=500 μm). **B**: Small interfering RNA–targeting tissue factor (TF-siRNA)-2 and vascular endothelial growth factor (VEGF)-siRNA decreased the total number of branching points compared with the blank control (*p<0.001). Data are shown as mean±standard deviation (SD) from three independent experiments.

The treatment of TF-siRNA-2 led to an incomplete and sparse network of tubes in Matrigel under LPS stimulation for 12 h, while HUVECs subjected to blank control treatment formed extensive and enclosed tube networks (Figure 5). Network formation, as judged by the number of branching points counted, was decreased by 47.4% and 59.4% following treatment with the TF-siRNA-2 and VEGF-siRNA, respectively, compared with the blank control (p<0.001). The difference in the number of branching points between the blank control and NS-siRNA–treated group was not statistically significant.

## Discussion

In this study, we established various in vitro models to test the efficacy of TF-siRNA in the suppression of NV. Our data have shown that LPS stimulation increased TF expression in HUVECs. The most efficient TF-siRNA from the tested candidates was able to substantially reduce the mRNA and protein expression of TF. MTT and wound-healing assays showed that the TF-siRNA inhibited the proliferation and migration of HUVECs, as well as tube formation. To the best of our knowledge, this is the first application of TF-siRNA in an in vitro model of NV.

Evidence has shown that TF plays an important role in tumor growth, angiogenesis, tumor invasion, and metastasis [39-41]. TF-targeted therapy has been proposed as a promising strategy for treating cancer patients. It has been reported that TF-siRNA can inhibit tumor growth and metastasis in vitro and in vivo [36], as well as suppressing the expression of TF in porcine neonatal islet cell clusters in vitro [42]. The mechanism underlying the tumor suppression by TF-siRNA might contribute to the apoptosis triggered by TF inhibition [43], which is mediated by blockage of the Erk MAPK and PI3K/Akt signal pathways [36], induction of JNK, and/or suppression of matrix metalloproteinases [43].

The progression of NV in ocular tissue is a multistep process similar to that of tumors. It involves many genetic and environmental risk factors, including oxidative stress and inflammation. Under pathological conditions, such as diabetic retinopathy and retinopathy of prematurity, the retina increases the production of angiogenic stimulators and reduces the production of angiogenic inhibitors, disrupting the balance between the positive and negative regulators of angiogenesis [44]. TF can initiate intracellular signaling and promote inflammation and angiogenesis. It has been hypothesized that sustained overproduction of TF by retinal cells during inflammation promotes retinal NV in several neovascular eye diseases [24].

We designed three pairs of TF-siRNAs for our studies using a web-based program according to Vert’s model [45]. The selected siRNAs sequences are different from those which have been reported [36,42,43]. The correct choice of siRNA is one of the most crucial steps in its reliable use. The parameters used in our design include hairpin potential formation, stability profiles, energy features, and the mRNA secondary structure.

Our findings clearly demonstrate that TF plays a crucial role in NV development. This is the first study to apply TF-siRNA as a means of controlling NV. We conclude that TF-siRNA may be a potential tool for CNV therapy, and might be extended to other NV-related diseases with high levels of TF expression as well. Although normal retinal cells do not express TF at detectable levels in physiological conditions [25], implying less hazardous consequences of TF suppression in comparison with VEGF blockage, further experiments remain necessary to determine whether detrimental side effects could appear after the local administration of TF-siRNA in vivo.
